# Thermoelectric tourniquet–assisted thermotherapy and cryotherapy for pain, regional blood flow, and satisfaction with intravenous injections among hospitalized patients in Korea: a randomized controlled trial

**DOI:** 10.7586/jkbns.24.027

**Published:** 2024-11-26

**Authors:** Seon-Mi Lee, Myung-Haeng Hur

**Affiliations:** 1 Daejeon Eulji University Hospital, Daejeon, Korea; 2 College of Nursing, Eulji University, Uijeongbu, Korea

**Keywords:** Cryotherapy, Pain, Regional blood flow, Hyperthermia, induced, Tourniquets

## Abstract

**Purpose:**

Intravenous injections are a common invasive procedure for delivering fluids or medications. Although patients experience pain from needle insertion, this discomfort is often overlooked due to its short duration and perceived insignificance. This study aimed to assess the effect of applying cryotherapy and thermotherapy using a flexible thermoelectric device on relieving the pain caused by intravenous injections.

**Methods:**

This randomized controlled trial utilized the modified thermoelectric element (M-TEE) tourniquet, which improves heat and cold transfer through a flexible TEE. Participants were hospitalized adults who had an 18-gauge angiocatheter inserted for surgery. The M-TEE tourniquet was applied 10-12 cm above the injection site, providing thermotherapy (40°C-45°C) or cryotherapy (0°C-10°C) to the respective groups. The control group received no temperature treatment. Pain, peripheral oxygen saturation, pulse rate, and regional blood flow were measured. Post-injection, satisfaction surveys were conducted with participants and practitioners.

**Results:**

There was a significant difference in pain perception among the three groups (F = 3.38, *p* = .041), with the cryotherapy group reporting less pain than the control group (*p* = .036). Regional venous blood flow significantly increased during thermotherapy (F = 5.99, *p* = .004), although regional arterial flow remained unchanged. Participant satisfaction differed across groups (F = 3.26, *p* = .046), with higher satisfaction in the cryotherapy group than in the control group (*p* = .040). Nurses’ satisfaction also varied significantly (F = 24.14, *p* < .001) across the groups.

**Conclusion:**

The M-TEE tourniquet effectively reduces intravenous injection pain and increases patient and practitioner satisfaction, particularly with cryotherapy.

## INTRODUCTION

Venipuncture for intravenous injection occupy an important position among the invasive procedures performed by nurses [1]. Venipuncture is often described as difficult for nurses and painful for patients [2]. If the pain experience due to venipuncture is repeated, the patient may feel fear of injections, which increases anxiety about future treatment, and if the patient does not actively participate in treatment due to pain, recovery may be delayed [3]. Most patients experience pain when the needle pierces the skin. Some patients may notice more pain and experience vagal reflexes, or it may lead to low blood pressure and syncope [4]. Therefore, continued research is needed to reduce patients' pain and anxiety related to venipuncture pain.

Previous research has consistently sought to identify methods to reduce the pain and stress associated with IV procedures. Pharmacological interventions, such as the application of topical anesthetics like lidocaine ointment or patches, have shown effectiveness in reducing pain during venipuncture [5,6]. Similarly, the application of Eutectic Mixture of Local Anesthetics (EMLA cream), consisting of 2.5% lidocaine and 2.5% prilocaine, has demonstrated significant pain-reducing effects [7]. However, the use of topical anesthetics may result in side effects such as hypersensitivity, skin erythema, swelling, and itching, warranting cautious application [6]. Additionally, the costs associated with these medications can be a financial burden for patients.

Non-pharmacological interventions, including cryotherapy [8] and thermotherapy [9], have also been employed to alleviate pain during venipuncture. Other methods such as music therapy [10], aromatherapy [11], distraction techniques using tablet video [12], and virtual reality content [13,14] have also been explored. Among these, thermotherapy is particularly noteworthy for its effectiveness as a nursing intervention for pain management [15]. Thermotherapy functions by enhancing nerve conduction, stimulating nerve endings, or blocking pain transmission through the gate control theory, thereby providing anesthetic and sedative effects. The relaxation induced by thermotherapy offers comfort, and its vasodilatory effect is particularly useful in alleviating pain associated with muscle spasms due to ischemia [16]. Research on thermotherapy has shown its effectiveness in reducing subjective pain during arteriovenous fistula needle insertion in hemodialysis patients [17] and in decreasing the time required for injections, the number of attempts, and increasing the success rate of first attempts in pediatric emergency patients [18]. Cryotherapy is a representative non-pharmacological intervention that reduces pain and swelling following tissue damage and suppresses inflammatory responses [19]. Cryotherapy induces vasoconstriction, which decreases local cellular metabolism and inhibits inflammatory and purulent processes, thereby preventing the accumulation of excess fluid in the injured tissue that could exacerbate pain [16]. Previous studies have shown that cryotherapy using an ice pack for 5~10 minutes is effective in reducing pain during venipuncture [20]. Although thermotherapy and cryotherapy are well-established non-pharmacological interventions for pain relief, there is limited evidence supporting their efficacy specifically for needle insertion pain. Furthermore, the inconvenience associated with the application methods of these therapies has restricted their widespread use during intravenous procedures. To promote broader application, it is necessary to address and mitigate the inconveniences related to the application methods of cryotherapy and thermotherapy.

Thermoelectric devices, which convert thermal energy directly into electrical energy and vice versa, are employed in various fields such as household appliances (refrigerators, dehumidifiers and water purifiers), computer systems (CPU cooling), automotive climate control seats, and industrial applications (waste heat generators, remote power generation) due to their excellent heating and cooling properties [21]. A pioneering study in South Korea developed a thermoelectric element band (TEE Band), enabling the convenient application of cryotherapy and thermotherapy using a thermoelectric device, and demonstrated its effectiveness in reducing pain during venipuncture [22].

The TEE Band, designed as a wristband, facilitates cryotherapy or thermotherapy during venipuncture through a button-controlled mechanism. It was found to be effective in pain relief when applied to the wrist during venipuncture while a tourniquet was used on the upper arm. The study recommended extending the application time of the band to maintain its effect during IV procedures [22]. Subsequently, the TEE tourniquet was developed, integrating flexible TEEs with a tourniquet and a temperature control device. This device proved significantly effective in pain relief, especially with thermotherapy, and both patients and practitioners reported high satisfaction. Recommendations included increasing the temperature plate area for more effective therapy application [23].

In this study, the modified TEE (M-TEE) tourniquet was developed by enlarging the plate area of the TEE tourniquet. The objective was to assess the effects of the M-TEE tourniquet's thermotherapy and cryotherapy on pain and regional blood flow during IV injections in hospitalized patients. The study also aimed to measure changes in regional blood flow due to thermotherapy and cryotherapy. Thermotherapy dilates blood vessels and increases regional blood flow, increasing the visibility of veins and thus increasing the success rate of intravenous injections. Therefore, it was thought that it would help reduce the number of intravenous injection attempts and pain, and the intention was to check data related to the actual regional blood flow rate. This investigation seeks to evaluate the M-TEE tourniquet’s impact on pain and regional blood flow during IV procedures, providing foundational data for improving pain management techniques in hospitalized patients requiring an 18-gauge angiocatheter for surgery.

## METHODS

### 1. Study design

**Figure 1.**
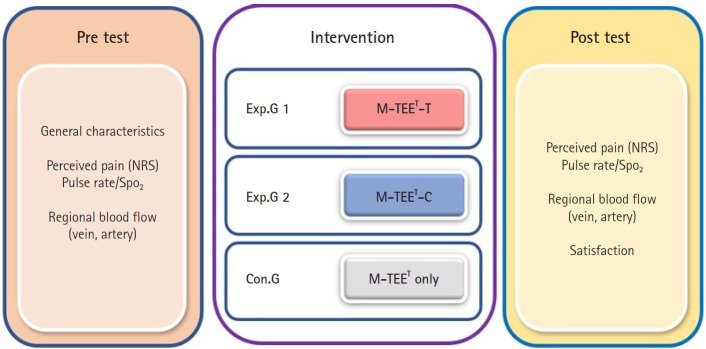
Study design. Exp. G = Experimental group; Con. G = Control group; M-TEE^T^-T =  Modified thermoelectric element tourniquet-thermotherapy group; M-TEE^T^-C = Modified thermoelectric element tourniquet-cryotherapy group; M-TEE^T^ only = Modified thermoelectric element tourniquet group; NRS = Numeric rating scale; Spo_2_ = Saturation of percutaneous oxygen.

This study employs a randomized controlled trial (RCT) to assess the effects of thermotherapy and cryotherapy using the M-TEE tourniquet on pain, regional blood flow, and patient satisfaction during 18-gauge angiocatheter insertion for surgical preparation. The design includes two experimental groups and one control group, as illustrated in Figure 1.

### 2. Participants

The study involved adults admitted to Daejeon Eulji Medical Center from February 6 to February 28, 2023, who required 18-gauge angiocatheter insertion for surgical preparation. Most of the participants were patients hospitalized for gynecology and otolaryngology surgery, and there were no restrictions on the injection site for surgery. Inclusion criteria were adults aged 20 to 65 years, who consented to participate, could communicate, and had prior experience with intravenous injections within the last six months. To exclude the influence of cognitive ability and chronic diseases on the study results, an age limit of 65 years or younger was set. Exclusion criteria included those undergoing pain relief treatments (e.g., analgesics, sedatives) affecting pain perception, individuals sensitive to heat or cold, and those with mental disorders impairing judgment.

**Figure 2.**
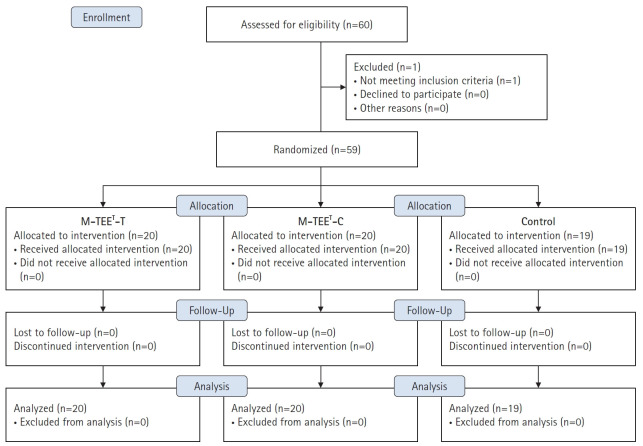
Flow diagram. M-TEE^T^-T = Modified thermoelectric element tourniquet-thermotherapy group; M-TEE^T^-C = Modified thermoelectric element tourniquet-cryotherapy group; Control = Control group.

Sample size was determined based on a prior study [24] utilizing pain relief methods during venipuncture. Using G*Power 3.1.9.7 for repeated measures analysis of variance (ANOVA) with an effect size of 0.25, significance level of 0.05, power of 0.9, three groups, three measurements, and a correlation coefficient of 0.4, the required sample size was 54. Including a 10% dropout rate, 60 participants were recruited, with 20 assigned to each group: thermotherapy, cryotherapy, and control. One participant in the control group was excluded for lacking recent intravenous injection experience, resulting in 20 participants in the thermotherapy group, 20 in the cryotherapy group, and 19 in the control group, as shown in Figure 2.

Participants were recruited via a convenience sample announced on the hospital ward bulletin board. Random assignment to groups was achieved using Excel randomization functions and coded recruitment order to minimize allocation bias. Participants were unaware of their group assignment, and the two research assistants measuring dependent variables were blinded to this information, although complete blinding was not feasible due to the study's nature. Randomization results were sealed in envelopes and kept in a secure box, with participants sequentially allocated according to envelope order.

### 3. Instruments

#### 1) Pain assessment

Pain was measured by assessing both the perceived pain and physiological indicators.

① Perceived pain: The perceived pain during injection was measured using the numeric rating scale, which ranges from 0 (no pain) on the left end to 10 (worst pain imaginable) on the right. Participants marked their perceived pain level before, during, and after the intravenous injection, with higher scores indicating more severe pain.

② Observed oxygen saturation and pulse rate (physiological response): Oxygen saturation and pulse rate were measured using a pulse oximeter (Pulse oximeter, 32MX, Nellcor, Covidien, USA) placed on the finger of the arm opposite to the injection site.

#### 2) Regional blood flow measurement

Regional blood flow at the intravenous injection site was measured using the SmartDop45 Doppler flowmeter (NICO Corporation, Koven Technology, Winnipeg, Manitoba, Canada), a compact, portable device. Measurements, in cm/sec, were taken from both regional veins and arteries. To assess the effects of thermotherapy and cryotherapy, baseline regional blood flow was measured on the opposite arm from the injection site. After the intravenous injection, an M-TEE tourniquet was applied 10–12 cm above the site of baseline measurement, ensuring close skin contact without occluding regional blood flow. Post-application regional blood flow in the vein and nearby artery was measured during thermotherapy or cryotherapy. Comparisons were made between baseline and post-application regional blood flow measurements on the arm opposite to the injection site.

The SmartDop45 Doppler is a portable bidirectional vascular Doppler with an Liquid Crystal Display waveform display and integrated printer. It emits ultrasound waves to measure frequency shift from moving red blood cells, providing data on blood flow direction and velocity [25]. Blood flow can be calculated using the following equation, where V represents blood flow (m/sec), c is the speed of sound (m/sec), fd is the Doppler frequency shift (Hz), ft is the transducer frequency (Hz), and θ is the angle between the blood flow and the beam (Doppler angle) [26].


$$V=\frac{f_{d}c}{2f_{t}\cos\theta }$$

A larger Doppler angle reduces measurement accuracy, thus, angles of 60 degrees or less are recommended for more precise results [26]. There are no standard reference values for blood flow; thus, only relative changes are observed.

#### 3) Satisfaction with M-TEE tourniquet application

① Participant satisfaction: This was measured using a modified questionnaire based on previous studies (Cronbach’s α = .87) [22]. The questionnaire, consisting of nine items measured on a 5-point Likert scale, assessed satisfaction with pain reduction, overall satisfaction, and willingness to reuse. Higher scores indicated greater satisfaction. The reliability of the instrument in this study was Cronbach’s α = .88. Detailed questions are in Supplement 1.

② Nurse satisfaction: The satisfaction of practitioners with the application of the M-TEE tourniquet during intravenous catheterization was evaluated using a revised questionnaire based on a previous study [22]. The revised tool incorporated additional items assessing vein dilation, the ease of injection, and the level of patient pain observed by the practitioner, resulting in a total of eight items. Each item was rated on a 5-point Likert scale ranging from "Strongly Disagree" to "Strongly Agree," with higher scores indicating greater satisfaction. The reliability of the instrument in this study was confirmed with a Cronbach’s α of .86. Detailed questions are in Supplement 2.

### 4. Experimental intervention

This study employed thermotherapy and cryotherapy during intravenous (IV) catheterization using a M-TEE tourniquet, which features two 5.5 cm × 3.5 cm plates.

#### 1) M-TEE tourniquet

A TEE generates electromotive force (emf) from temperature differences or induces a temperature change when an external emf is applied, making it suitable for heating or cooling [27,28]. During peripheral venipuncture, it is essential to use a tourniquet over the puncture site to enhance venous distension [29]. In order to expand the area that provides heating and cooling of the previously developed TEE tourniquet (including one flexible thermoelectric plate), a nursing professor, a doctor of materials engineering, and a person in charge of flexible TEE development who participated in the development discussed together. The M-TEE tourniquet was upgraded to include two flexible thermoelectric plates (5.5 cm × 3.5 cm), based on South Korean patents (No. 10-1989908, 10-1829709, 10-1689308). This modified tourniquet, applied 10~12 cm above the IV site, was used in place of a conventional tourniquet, providing either thermotherapy or cryotherapy before IV catheterization.

#### 2) M-TEE tourniquet temperature and application duration

Thermotherapy, which involves heat application, increases regional blood flow and relaxes muscles, aiding pain relief [30]. Cryotherapy, applied to the skin or deeper tissues, causes vasoconstriction, reducing metabolism, muscle spasms, inflammation, and pain [31].

The M-TEE tourniquet offers three temperature settings for thermotherapy: approximately 36.6°C (stage 1), 41.4°C (stage 2), and 54.4°C (stage 3). For cryotherapy, temperatures are around 13.5°C (stage 1), 4.9°C (stage 2), and -2.6°C (stage 3). Safe temperature ranges are 40°C~45°C for thermotherapy and 0°C-10°C for cryotherapy [32-34]. Due to individual variability in skin sensitivity, discomfort was assessed for five seconds after initiating either thermotherapy or cryotherapy. Upon verifying temperature appropriateness, the area was continuously exposed to the specified temperature while being disinfected with an alcohol swab and preparing the Angiocatheter. This preparation took at least 10 seconds. If discomfort occurred during temperature adjustment, the temperature was reduced to stage 1. The M-TEE tourniquet was positioned 10~12 cm above the planned injection site, and either thermotherapy or cryotherapy was applied during the IV injection. The duration of temperature application ranged from 10 to 30 seconds, depending on the success of the IV insertion.

#### 3) M-TEE tourniquet intervention

Participants, scheduled for 18 gauge angiocatheter insertion for surgery, were recruited. The M-TEE tourniquet was used as a tourniquet with thermotherapy or cryotherapy as per group assignment. Detailed procedures are in Supplement 3.

### 5. Data collection

Data was collected at Daejeon Eulji Medical Center from February 6 to February 28, 2023.

#### 1) Pre-approval procedure

After IRB approval, recruitment posters were displayed on hospital ward bulletin boards with departmental authorization.

#### 2) Research assistants' preparation

Two research assistants were trained: One nurse with 10 years of clinical experience, explained the study procedures, obtained written consent, and performed intravenous injections with the M-TEE tourniquet. Another nurse with nine years of clinical experience, measured peripheral oxygen saturation and pulse rate to ensure in measurements.

#### 3) Pre-measurement

Interested participants were informed about the study and consented. They completed a questionnaire on their general characteristics and pain from previous intravenous injections. Baseline pulse and oxygen saturation were recorded, and regional blood flow in veins and arteries at the opposite arm was measured.

#### 4) Experimental intervention

Research Assistant 1 applied an M-TEE tourniquet 10 to 12 cm above the injection site according to Fundamental Nursing Guidelines for intravenous injection [4]. Research Assistant 2 administered either thermotherapy (40℃-45℃) or cryotherapy (0℃-10℃) for five seconds before maintaining the temperature during injection. The reason it was applied five seconds before venipuncture was to check sensitivity to heat or cold and prevent side effects due to temperature application. Once the temperature was confirmed to be appropriate, venipuncture was performed while maintaining the temperature. The venipuncture site was selected as the cephalic vein on the wrist or the cephalic vein on the forearm. Areas where arteries and veins were palpable were preferentially selected for venipuncture and regional blood flow measurements. The control group received the M-TEE tourniquet without temperature control. Pulse and oxygen saturation were monitored during the injection.

#### 5) Post-measurement

After catheter insertion, the M-TEE tourniquet was removed and the catheter secured. Post-injection pulse rate and oxygen saturation were recorded. Regional blood flow measurements were repeated on the opposite arm, and participants completed a survey on pain and satisfaction. Research Assistant 1 also filled out a satisfaction survey regarding the tourniquet.

### 6. Data analysis

Data were analyzed using IBM SPSS Statistics 26.0 (IBM Corp., Armonk, NY, USA). General characteristics of the participants were assessed using frequency, percentage, mean, and standard deviation. Homogeneity tests were conducted using the χ^2^-test and ANOVA. The homogeneity of dependent variables across the thermotherapy, cryotherapy, and control groups was verified using ANOVA. Pain and satisfaction levels before, during, and after intravenous injection with the M-TEE tourniquet were analyzed using ANOVA, followed by Bonferroni post-hoc analysis for significant results. Changes before and after the intervention were evaluated using repeated measures ANOVA. If sphericity was violated, Wilks' Lambda multivariate tests were conducted. The reliability of the satisfaction tool for the M-TEE tourniquet was assessed using Cronbach’s alpha. Partial eta-squared (partial η^2^) was calculated to assess the influence of independent variables over time.

### 7. Ethical considerations

The researcher completed training on ethical research involving human subjects in May 2022. The study plan was approved by the Institutional Review Board of Eulji University Hospital (IRB No.: EMC 2022-06-004-002) and registered with the Clinical Research Information Service (CRIS) (KCT0008538).

Prior to data collection, approval was obtained from the institution and nursing department, and recruitment notices were posted. Participants were informed of the study's purpose and methods, and consent was obtained through signed forms. Participants were also informed of their right to withdraw at any time. As the study involved the use of a temperature-controllable band that contacts the skin, potential side effects (e.g., contact dermatitis) and their management were explained. Participants in all groups received a 5,000 KRW cash incentive after the intervention.

To ensure the protection of personal information, all collected data were assigned unique identifiers and were not used for any purposes outside of this study. Participant information was securely stored in a locked cabinet, and all data will be retained for three years following the conclusion of the study, after which they will be destroyed by shredding.

## RESULTS

### 1. Verification of homogeneity in participants' general characteristics and dependent variables

A total of 59 participants were included in this study, with 20 in the thermotherapy group, 20 in the cryotherapy group, and 19 in the control group. The results of the homogeneity analysis regarding the general characteristics and dependent variables of the three groups are presented in Table 1. There were no significant differences among the three groups in general characteristics and baseline of pain, regional venous and arterial flow; therefore, the three groups were considered to be homogeneous.

### 2. Effects of the M-TEE tourniquet on pain, oxygen saturation, pulse rate, and regional blood flow during intravenous injection

#### 1) Pain

**Figure 3.**
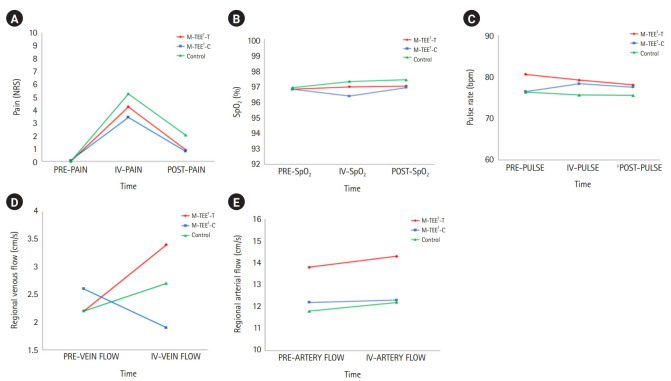
Comparison of perceived pain (3-A), SpO_2_ (3-B), pulse rate (3-C), regional venous blood flow (3-D), regional arterial blood flow(3-E) among three group. M-TEE^T^-T = Modified thermoelectric element tourniquet thermotherapy group; M-TEE^T^-C = Modified thermoelectric element tourniquet cryotherapy group; Control = Control group; NRS: Numeral rating scale; Past pain = Past injection pain, pain experienced during intravenous injection within 6 months; Pre pain = pre-injection pain, pain felt before intravenous injection; IV pain = Pain during intravenous injection, pain felt during intravenous injection; Post pain = Pain after intravenous injection; SpO_2_ = Saturation of percutaneous oxygen; Pre SpO_2_ = Saturation of percutaneous oxygen before intravenous injection; IV SpO_2_ = Saturation of percutaneous oxygen during intravenous injection; Post SpO_2_ = Saturation of percutaneous oxygen after intravenous injection; Pre Pulse = Pulse rate before intravenous injection; IV Pulse = Pulse rate during intravenous injection; Post Pulse = Pulse rate after intravenous injection; Bpm = Beats per min; Pre vein flow = Regional venous blood flow before intravenous injection; IV vein flow = Regional venous blood flow during intravenous injection; Pre artery flow = Regional arterial blood flow before intravenous injection; IV artery flow = Regional arterial blood flow during intravenous injection.

The effects of the M-TEE tourniquet on pain during intravenous injection are summarized in Table 2. During 18-gauge angiocatheter insertion, pain scores showed significant differences among groups (F = 3.38, *p* = .041), with post-hoc analysis indicating significantly lower pain in the cryotherapy group compared to the control group (*p* = .036). The changes in pain before, during, and after intravenous injection were analyzed using repeated measures ANOVA. The Mauchly's test of sphericity was satisfied (W = .94, *p* = .183), indicating a significant difference over time (F = 140.76, *p* < .001), a significant difference among the three groups (F = 3.95, *p* = .025), and a significant interaction between time and group (F = 2.52, *p* = .045) (Figure 3). The partial eta squared value for the effect size of the M-TEE tourniquet application by time and group was 0.08.

#### 2) Oxygen saturation

Oxygen saturation was measured before, during, and after intravenous injection, and there were no significant differences between the three groups (F = 0.02, *p* = .981).

#### 3) Pulse rate

Pulse rate was measured before, during, and after the intravenous injection, and there were no significant differences between the three groups (F = 0.78, *p* = .462) (Table 2, Figure 3).

#### 4) Regional blood flow

Before applying the M-TEE tourniquet, regional venous and arterial blood flow were measured from the same vein on the opposite arm of the planned intravenous injection site. Post-application regional venous blood flow, measured 10~12 cm above the same vein on the opposite arm, showing no significant differences (F = 2.17, *p* = .124). Comparisons of regional venous blood flow before and during M-TEE tourniquet application revealed no significant differences among the groups (Table 2). The change in regional venous blood flow before and during M-TEE tourniquet application was 1.16 ± 1.34 cm/s for the thermotherapy group, -0.77 ± 1.80 cm/s for the cryotherapy group, and 0.47 ± 2.13 cm/s for the control group. This difference was statistically significant (F = 5.99, *p* = .004) (Table 2, Figure 3). Post-hoc analysis indicated a significant difference between the thermotherapy and cryotherapy groups (*p* = .004). Repeated measures ANOVA, using Wilks’ lambda due to Mauchly’s test failure (W = 1.00, *p* < .001), revealed no significant changes over time (F = 1.54, *p* = .220) or differences among groups (F = 0.38, *p* = .689), but a significant interaction between time and group (F = 5.99, *p* = .004). The partial eta squared for the effect size of the M-TEE tourniquet on regional venous blood flow in relation to time and group was 0.18.

There were no significant differences in regional arterial blood flow were observed before and during M-TEE tourniquet application (F = 0.03, *p* = .973) (Table 2, Figure 3).

#### 5) Participant satisfaction

The participant satisfaction scores for M-TEE tourniquet application during intravenous injection were found to be significantly different between the three groups (F = 3.26, *p* = .046), with post-hoc analysis revealing a significant difference between the cryotherapy group and the control group (*p* = .040) (Table 2).

#### 6) Nurse satisfaction

There was a significant difference in nurse satisfaction scores between groups (F = 24.14, *p* < .001), and post hoc analysis showed that satisfaction was higher in both the thermotherapy and cryotherapy groups compared to the control group (*p* < .001) (Table 2).

## DISCUSSION

This study evaluated the efficacy of the M-TEE tourniquet, a device with a plate area twice that of the standard TEE tourniquet, for thermotherapy and cryotherapy during intravenous catheter insertion. In the experimental group, participants received thermotherapy or cryotherapy for 10 seconds prior to venipuncture, while the control group underwent the procedure without temperature treatment. The choice of a 10-second duration aligns with findings from a Cochrane systematic review on vapocoolants for pain management during IV cannulation, which indicated that cold spray applications of 5~10 seconds yield significant pain relief [35].

Key variables included historical reports of injection pain, current pain levels during the injection, oxygen saturation, pulse rate, and both regional arterial and venous blood flow. The thermoelectric technology used in the M-TEE tourniquet, designed to create rapid temperature changes through electrical stimulation, was previously adapted into a band format [22] before this tourniquet adaptation [23]. In this study, the M-TEE tourniquet, which features an expanded surface area, was utilized to apply cryotherapy and thermotherapy. The results demonstrated a significant difference in pain perception among the three groups, with the cryotherapy group experiencing notably less pain.

Earlier studies have explored diverse approaches to injection pain relief using both cryotherapy and thermotherapy For instance, ice massage on the palm during arteriovenous fistula puncture in hemodialysis patients significantly reduced pain [36]. Applying ice packs for 10 minutes at cannulation sites also reduced arteriovenous fistula pain [37], while an RCT using vibration and cold gel packs during IV catheterization reported reduced pain in the intervention group [24].

Additionally, when measuring injection pain in participants undergoing peripheral intravenous cannulation, no significant difference was found between the experimental group that received local warming and the control group that did not [35]. In contrast, a study that assessed pain, anxiety, and vein assessment after applying either a hot or cold application to the insertion site for one minute prior to peripheral venous catheter insertion found that the groups receiving local hot and cold applications reported lower pain and anxiety levels compared to the control group [3]. Specifically, while pain levels were reduced in both the hot and cold application groups compared to the control group, there was no significant difference between the two experimental groups. However, anxiety levels were significantly lower in both the hot application and cold application groups compared to the control group, with the hot application group exhibiting significantly lower anxiety levels than both the cold application group and the control group [3].

Previous studies have reported significant differences in pain relief associated with cryotherapy, ice massage [36] and utilizing ice packs [37], as well as with thermotherapy involving hot packs [18], hand warmers [19] and hot packs containing silicate gel [32]. Additionally, both cryotherapy and thermotherapy proved effective when using a TEE band [22] and TEE tourniquet [23]. In research where hot packs were applied for 10 minutes and 20 minutes [32], both experimental groups exhibited significantly lower pain levels compared to the control group, and due to the minimal differences between the two experimental groups, it was concluded that a 10-minute application of hot packs could be effectively utilized without adverse effects. Similarly, another study applying a hot pack for 15 minutes demonstrated a reduction in pain associated with needle cannulation [17]. The time required for intravenous injections was shorter in the experimental group receiving heat therapy, with this difference being statistically significant [18].

However, in a RCT comparing the application of a TEE band on the wrist that provided heat therapy, cold therapy, and thermo-grill illusion therapy against a control group that received no treatment [22], the post-intervention pain scores indicated no significant differences among the four groups. Although the pain levels for the cryotherapy group were notably lower compared to pre-treatment measurements, statistical significance was not established among the four groups. Conversely, a study that modified the TEE band into a TEE tourniquet [23] reported that thermotherapy using the TEE tourniquet significantly reduced injection pain and also resulted in lower stress levels.

Previous studies have primarily focused on alleviating injection pain in pediatric patients or those with arteriovenous fistulas. However, this study broadens the scope by examining the effects of cryotherapy and thermotherapy on adult patients undergoing surgery. Most of the participants in the study were otolaryngology patients and obstetrics and gynecology patients, most of whom had no underlying diseases and were discharged about 2-3 days after surgery. They were subjects who could reduce differences in vascular conditions depending on disease and intravenous injection pain depending on vascular conditions. This expansion of the target population is a notable contribution of the study. Nevertheless, discrepancies were observed in the effectiveness of temperature therapies. In prior research applying thermotherapy or cryotherapy in the form of tourniquets, thermotherapy alone was found to be effective for pain relief [23].

In contrast, the current study, which utilized the M-TEE tourniquet with an expanded temperature application area, found that only cryotherapy was effective. This variation is likely influenced by factors such as application time, area size, and temperature. In this study, the application time ranged from 10 to 30 seconds, while previous studies reported application times from 10 to 60 seconds. The plate area in prior research was 19.3 cm^2^, compared to 38.5 cm^2^ in this study. There is limited data on the application area size for ice packs or heating pads. The variation in findings across studies suggests that while thermoelectric devices demonstrate potential for pain management during injections, the relative efficacy of thermotherapy versus cryotherapy remains inconclusive. These discrepancies may arise from differences in methodological approaches, including the duration of application, the surface area treated, specific temperature settings, and patient-specific factors. To ensure the effective integration of thermoelectric devices into clinical practice, further research is essential to develop standardized guidelines that optimize these variables for pain relief. One possible reason for the differences in study outcomes may be the presence of multiple confounding variables in intravenous catheter insertion. Previous studies have indicated that factors such as the nurse’s age, level of experience, specialty, and proficiency in intravenous skills, along with patient-related and IV-related variables, can all have diverse influences on the outcomes [35].

No significant differences in SpO_2_ or pulse rates were observed among groups. A similar study on arteriovenous fistula patients found no significant changes in physiological stress indicators like blood pressure and pulse with ice application [37]. The absence of significant changes in this study suggests that the pain levels experienced by adult participants may not have been high enough to alter SpO_2_ or pulse.

In this study, regional blood flow was selected as an outcome variable because hot application is generally known to promote vasodilation, and previous research [3] has shown that vein visibility increased in the hot application group during vein assessment. Therefore, this study measured both regional venous and arterial blood flow. While no significant differences were observed in regional arterial blood flow before and after the intervention, a significant difference was found among the three groups regarding regional venous blood flow, with the thermotherapy group showing a marked increase.

In studies measuring regional blood flow during the application of thermotherapy, it was found that regional venous blood flow significantly increased in subjects receiving thermotherapy. This aligns with the known effects of heat and cold, where increased temperature causes vasodilation, and decreased temperature causes vasoconstriction [16]. However, the absence of significant differences in regional arterial blood flow among the three groups suggests that the effects of temperature did not extend to the arteries. This effect is considered beneficial as it may enhance the ease of intravenous injection for nurses, thereby offering positive implications for clinical practice.

Regarding satisfaction, participants in the cryotherapy group reported higher satisfaction, likely due to effective pain relief. For nurses, satisfaction levels increased in both cryotherapy and thermotherapy groups, with thermotherapy showing a greater impact. The increased venous flow from thermotherapy likely contributed to easier venous cannulation, positively affecting nurse satisfaction.

This study faced limitations in participant blinding, as randomization did not prevent participants from identifying their group upon receiving the intervention. Additionally, nurses were likely aware of each participant’s group during device activation, limiting the study's double-blind nature—a core aspect of RCTs. Although it was not possible to completely blind the group to which participants were assigned, the nurse tried to remain neutral to ensure accurate research results, refrained from mentioning the intervention, and tried to focus on investigating the items felt by the participants.

## CONCLUSION

The M-TEE tourniquet was effective in reducing injection pain and enhancing patient satisfaction in the cryotherapy group, with higher practitioner satisfaction noted in the thermotherapy group. Nonetheless, evidence regarding optimal application duration, surface area, and temperature remains insufficient, calling for further studies to establish standardized guidelines for effective clinical use of thermoelectric devices in pain relief. The M-TEE tourniquet demonstrates potential as an effective and convenient tool for managing injection pain. Although initial equipment costs may be incurred, the device's ease of use, simple training requirements, and effectiveness in alleviating patient pain make it a worthwhile investment. This study highlights its nursing significance, as the M-TEE tourniquet allows for easy implementation of pain-relief interventions. By expanding the temperature application area, this study contributes to optimizing pain management and improving patient and practitioner satisfaction during IV procedures.

## Figures and Tables

**Table 1.** Homogeneity Test of General Characteristics among the Three Groups (N = 59)

Characteristics	Categories	M-TEE^T^-T (n = 20)	M-TEE^T^-C (n = 20)	Control (n = 19)	χ^2^ or F	*p*
Sex	Men	8 (40.0)	7 (35.0)	7 (36.8)	0.11	.947
	Women	12 (60.0)	13 (65.0)	12 (63.2)		
Age (yr)		45.90 ± 13.35	47.25 ± 13.19	49.84 ± 14.44	0.42	.661
Height (cm)		165.00 ± 8.77	163.70 ± 7.35	163.58 ± 8.95	0.18	.840
Weight (kg)		60.70 ± 11.09	67.70 ± 13.87	64.95 ± 14.55	0.28	.758
Exercise	Fewer than 3 times a week	14 (70.0)	16 (80.0)	13 (68.4)	0.79	.674
	3 or more times a week	6 (30.0)	4 (20.0)	6 (31.6)		
Smoking	Yes	4 (20.0)	8 (40.0)	3 (15.8)	3.48	.175
	No	16 (80.0)	12 (60.0)	16 (84.2)		
Medical history	Surgical	10 (50.0)	11 (55.0)	13 (68.4)	1.14	.565
	Gynecological	10 (50.0)	9 (45.0)	6 (31.6)		
Drug	Yes	0 (0.0)	0 (0.0)	0 (0.0)	.	.
(Pain control)	No	20 (100.0)	20 (100.0)	19 (100.0)		
Past pain^†^ (NRS)		4.55 ± 2.42	4.35 ± 2.45	5.26 ± 1.56	0.93	.401
Regional vein flow (cm/s)		2.24 ± 2.30	2.65 ± 2.25	2.20 ± 2.01	0.25	.778
Regional artery flow (cm/s)		13.77 ± 8.70	12.24 ± 5.31	11.85 ± 5.14	0.47	.630

Values are presented as the mean ± standard deviation or n (%). M-TEE^T^-T = Modified thermoelectric element tourniquet thermotherapy group; M-TEE^T^-C = Modified thermoelectric element tourniquet cryotherapy group; Control = Control group; NRS = Numeral rating scale. ^†^Pain experienced during intravenous injection within the past 6 months.

**Table 2.** Comparison of Perceived Pain, SpO_2_, Pulse Rate, Regional Blood Flow, Satisfaction among the Three Groups (N = 59)

Variables		M-TEE^T^-T^a^ (n = 20)	M-TEE^T^-C^b^ (n = 20)	Control^c^ (n = 19)	F (*p*)	F (*p*)^†^
Pain (NRS)	Past pain^‡^	4.55 ± 2.42	4.35 ± 2.45	5.26 ± 1.56	0.93 (.401)	Time
	Pre-injection pain	0.00	0.08 ± 0.34	0.00	0.97 (.384)	140.76 (< .001)
						Group
						3.95 (.025)
	IV pain^§^	4.25 ± 2.45	3.43 ± 2.07	5.26 ± 2.08	3.38 (.041)	G*T
					b < c*	2.52 (.045)
	Post-injection pain	0.90 ± 1.62	0.78 ± 1.64	2.05 ± 2.07	3.02 (.057)	
SpO_2_ (%)	Pre-injection SpO_2_	96.80 ± 2.09	96.80 ± 1.54	96.89 ± 1.52	0.02 (.981)	Time
						0.93(.399)
						Group
						0.38 (.688)
	IV SpO_2_	96.95 ± 1.93	96.60 ± 1.67	97.26 ± 1.63	0.70 (.500)	G*T
	Post-injection SpO_2_	97.00 ± 1.86	96.90 ± 1.45	97.37 ± 1.54	0.45 (.643)	0.42 (.795)
Pulse rate (bpm)	Pre-injection pulse	80.55 ± 12.66	76.40 ± 11.36	76.26 ± 12.72	0.78 (.462)	Time
						1.34 (.270)
						Group
						0.46 (.633)
	IV pulse^∥^	79.15 ± 12.11	78.30 ± 11.35	75.58 ± 10.59	0.52 (.597)	G*T
	Post-injection pulse	78.00 ± 11.42	77.45 ± 11.48	75.47 ± 10.49	0.28 (.761)	1.61 (.177)
Regional vein flow (cm/s)	Pre-injection regional vein flow	2.24 ± 2.30	2.65 ± 2.25	2.20 ± 2.01	0.25 (.778)	Time
						1.54 (.220)
						Group
						0.38 (.689)
	IV regional vein flow	3.40 ± 1.98	1.88 ± 1.90	2.67 ± 2.93	2.17 (.124)	G*T
	Difference (V_2_-V_1_)	1.16 ± 1.34	-0.77 ± 1.80	0.47 ± 2.13	5.99 (.004)	5.99 (.004)
					a > b*	
Regional artery flow (cm/s)	Pre-injection regional artery flow	13.77 ± 8.70	12.24 ± 5.31	11.85 ± 5.14	0.47 (.630)	Time
						0.15 (.704)
						Group
						0.72 (.490)
	IV regional artery flow	14.26 ± 8.06	12.30 ± 4.94	12.18 ± 4.98	0.70 (.500)	G*T
	Difference (A_2_-A_1_)	0.49 ± 7.38	0.06 ± 3.80	0.34 ± 5.88	0.03 (.973)	0.03 (.973)
Patient satisfaction		30.30 ± 3.99	31.90 ± 2.75	28.42 ± 5.62	3.26 (.046)	
					b > c*	
Nurse satisfaction		29.95 ± 2.24	28.90 ± 2.77	24.32 ± 3.00	24.14 (< .001)	
					a,b > c**	

Values are presented as the mean ± standard deviation. M-TEE^T^-T = Modified thermoelectric element tourniquet thermotherapy group; M-TEE^T^-C = Modified thermoelectric element tourniquet cryotherapy group; Control = Control group; NRS = Numeral rating scale; SpO_2_ = Saturation of percutaneous oxygen; IV SpO_2_ = Saturation of percutaneous oxygen during intravenous injection; bpm = beats per min; IV regional vein flow(V_2_) = Regional venous blood flow during intravenous injection; Pre-injection regional artery flow(A_1_) = Regional arterial blood flow beforeintravenous injection; IV regional artery flow(A_2_) = Regional arterial blood flow during intravenous injection. ^†^Repeated measures analysis of variance; ^‡^Pain experienced during an intravenous injection within the past 6 months; §Pain during intravenous injection; ^∥^Pulse rate during intravenous injection. *p ≤ .05, Bonferroni; **p ≤ .01, Bonferroni.

## Data Availability

The data that support the findings of this study are available from the corresponding author upon reasonable request.

## Supplementary Materials

Supplement 1. Participant satisfaction

Supplement 2. Nurse Satisfaction

Supplement 3. Intervention Process
