# Supplementary material for: Thermoelectric tourniquet–assisted thermotherapy and cryotherapy for pain, regional blood flow, and satisfaction with intravenous injections among hospitalized patients in Korea: a randomized controlled trial
Source: J Korean Biol Nurs Sci. 2024 Nov 26;26(4):323–36. doi: 10.7586/jkbns.24.027 (PMC13268268; doi:10.7586/jkbns.24.027)
Supplement: Supplementary file 1 [file jkbns-24-027-Supplment-1.pdf]

Supplement 1. Participant satisfaction

This is what I felt during the intravenous injection. Among intravenous injections, please mark the answer that matches your opinion.

		Strongly Disagree	Disagree	Average	Agree	Strongly Agree
1	Injection pain was reduced compared to the previous injection.					
2	The next time I get the same injection, I will use the M-TEE tourniquet.					
3	I will also use the M-TEE tourniquet next time I get another injection (vaccination, etc.).					
4	I am satisfied with using the M-TEE tourniquet.					
5	I would recommend M-TEE Tourniquet to others.					
6	Stress decreased when receiving intravenous injections.					
7	M-TEE tourniquet is helpful for use in people with poor blood vessels.					
8	I would use the M-TEE tourniquet again even if it costs extra.					
9	I will speak positively to others about the M-TEE tourniquet.					
