# Supplementary material for: Thermoelectric tourniquet–assisted thermotherapy and cryotherapy for pain, regional blood flow, and satisfaction with intravenous injections among hospitalized patients in Korea: a randomized controlled trial
Source: J Korean Biol Nurs Sci. 2024 Nov 26;26(4):323–36. doi: 10.7586/jkbns.24.027 (PMC13268268; doi:10.7586/jkbns.24.027)
Supplement: Supplementary file 2 [file jkbns-24-027-Supplment-2.pdf]

Supplement 2. Nurse Satisfaction

This is what the nurse felt while injecting the patient intravenously. Please put a V in the box that matches your thoughts after intravenous injection.

	Question	Strongly Disagree	Disagree	Average	Agree	Strongly Agree
1	The use of M-TEE tourniquet was effective in relieving pain during intravenous injection in subjects.					
2	The use of the M-TEE tourniquet was helpful in administering intravenous fluids to participants.					
3	The use of the M-TEE tourniquet helped increase the participant's venous blood flow.					
4	The use of M-TEE tourniquet was helpful in identifying the subject's veins.					
5	I found the M-TEE tourniquet more convenient to use than the existing tourniquet.					
6	The M-TEE tourniquet caused less discomfort to the patient than the existing tourniquet.					
7	I feel positive about using the M-TEE tourniquet.					
8	I will recommend the use of M-TEE tourniquet to other nurses when administering intravenous injections.					
