# Supplementary material for: Thermoelectric tourniquet–assisted thermotherapy and cryotherapy for pain, regional blood flow, and satisfaction with intravenous injections among hospitalized patients in Korea: a randomized controlled trial
Source: J Korean Biol Nurs Sci. 2024 Nov 26;26(4):323–36. doi: 10.7586/jkbns.24.027 (PMC13268268; doi:10.7586/jkbns.24.027)
Supplement: Supplementary file 3 [file jkbns-24-027-Supplment-3.pdf]

Supplement 3. Intervention Process

	Process	Implementer	Author	Measuring Items
1	Research Assistant 1 explained the research procedures using an explanatory note and obtained written consent in the research consent form.	Research assistant 1	Reasearch participant	
2	Research Assistant 1 had the study subjects fill out a questionnaire about their general characteristics and the level of pain and stress (NRS) during intravenous injections experienced within the past 6 months.	Research assistant 1	Reasearch participant	General characteristics and intravenous injection pain and stress experienced within 6 months
3	Research Assistant 2 had the participant assume a comfortable position for receiving the injection, then connected a pulse oximeter to the finger of the arm opposite the injection site, and measured the pulse and oxygen saturation before the experimental treatment.	Research assistant 2	Research assistant 2	Pre-pulse, oxygen saturation
4	Research Assistant 2 measured venous blood flow and arterial blood flow in the blood vessel at the same location as the blood vessel scheduled for intravenous injection on the arm opposite to the injection site.	Research assistant 2	Research assistant 2	Preliminary blood flow measurements
5	Research Assistant 1 applied the M-TEE Tourniquet 10 to 12 cm above the vascular site where intravenous injection was scheduled to be performed.	Research assistant 1		

	Process	Implementer	Author	Measuring Items
6	Research Assistant 2 turned on the M-TEE Tourniquet temperature button appropriate for the assigned group.<br><br>· Thermotherapy group: HOT button, 2 levels (40~45℃), applied for a minimum of 10 seconds to a maximum of 30 seconds depending on the successful time of intravenous injection.<br><br>· Cryotherapy group: COLD button, level 2 (0~10℃) was applied for a minimum of 10 seconds to a maximum of 30 seconds depending on the success of intravenous injection.<br><br>· Control group: Temperature power was not turned on.	Research assistant 2		
7	Research Assistant 1 confirmed discomfort due to temperature for 5 seconds. If the participant had no discomfort due to temperature, the intravenous injection site was disinfected with an alcohol swab and then intravenous injection was performed using an 18-gauge Angiocatheter. Upon successful intravenous insertion, the M-TEE Tourniquet was removed.	Research assistant 1		

	Process	Implementer	Author	Measuring Items
	The moment Research Assistant 1 punctured the intravenous injection site, Research Assistant 2 checked and recorded the pulse and oxygen saturation during the injection.	Research assistant 1, 2	Research assistant 2	Pulse and oxygen saturation during injection
7	After successful intravenous injection, Research Assistant 1 passed saline solution to confirm patency and completed the intravenous injection by attaching a fixing tape. Research Assistant 2 checked and recorded the pulse and oxygen saturation after the intravenous injection at the end of the treatment.	Research assistant 1, 2	Research assistant 1, 2	Pulse and oxygen saturation after injection
8	Research Assistant 2 applied the M-TEE Tourniquet 10 to 12 cm above the blood vessel where prior blood flow was measured. At this time, it was applied to a degree that adhered closely to the skin but did not block blood flow, and thermotherapy or cryotherapy was applied. Venous blood flow was measured with the temperature applied starting 10 seconds after the temperature was applied. After measuring venous blood flow, blood flow was measured in arteries close to the veins.	Research assistant 2	Research assistant 2	Blood flow during injection

	To accurately measure blood flow, ultrasonic gel was applied to the participant's skin and the probe was moved to find the point where the blood flow wave on the blood flow meter screen was best measured. After holding the area for 5 seconds, press the probe botten to freeze the measured screen and then press the print button to print out the measurement results.	Research assistant 2		
	**Process**	**Implementer**	**Author**	**Measuring Items**
9	After all procedures were completed, Research Assistant 1 used a questionnaire to have participants fill out the level of pain and stress (NRS) they felt before, during, and after the intravenous injection and their satisfaction with the application of the M-TEE Tourniquet.	Research assistant 1	Research participants	Before, during, and after intravenous injection pain and stress/participant's Using M-TEE Tourniquet satisfaction
10	After completing all processes, Research Assistant 2 provided a stipend of 5,000 won to the research participants.	Research assistant 2		
11	Lastly, Research Assistant 1 filled out a questionnaire about the operator's satisfaction with the M-TEE Tourniquet felt during the intravenous injection process.	Research assistant 1	Research assistant 1	Satisfaction with using M-TEE Tourniquet
